# Innovative therapeutic strategy for B-cell malignancies that combines obinutuzumab and cytokine-induced killer cells

**DOI:** 10.1136/jitc-2021-002475

**Published:** 2021-07-16

**Authors:** Anna Dalla Pietà, Elisa Cappuzzello, Pierangela Palmerini, Annavera Ventura, Andrea Visentin, Giuseppe Astori, Katia Chieregato, Valentina Mozzo, Omar Perbellini, Maria Chiara Tisi, Livio Trentin, Carlo Visco, Marco Ruggeri, Roberta Sommaggio, Antonio Rosato

**Affiliations:** 1 Department of Surgery, Oncology and Gastroenterology, Immunology and Oncology Section, University of Padua, Padova, Italy; 2 Hematology and Clinical Immunology Unit, Department of Medicine, University of Padua, Padova, Italy; 3 Advanced Cellular Therapy Laboratory, Department of Hematology, San Bortolo Hospital of Vicenza, Vicenza, Italy; 4 Consorzio per la Ricerca Sanitaria (CORIS) of Veneto Region, Padova, Italy; 5 Veneto Institute of Oncology IOV - IRCCS, Padova, Italy; 6 Cell Therapy and Hematology, San Bortolo Hospital, Vicenza, Italy; 7 Department of Medicine, Section of Hematology, University of Verona, Verona, Italy

**Keywords:** combined modality therapy, hematologic neoplasms, immunotherapy, adoptive

## Abstract

**Background:**

Patients affected by aggressive B-cell malignancies who are resistant to primary or salvage chemoimmunotherapy have an extremely poor prognosis and limited therapeutic options. Promising therapeutic success has been achieved with the infusion of CD19 chimeric antigen receptor-T cells, but several limits still restrain the administration to a limited proportion of patients. This unmet clinical need might be fulfilled by an adoptive immunotherapy approach that combines cytokine-induced killer (CIK) cells and monoclonal antibodies (mAb) to the CD20 antigen. Indeed, CIK cells are an effector population endowed with antitumor activity, which can be further improved and antigen-specifically redirected by clinical-grade mAb triggering antibody-dependent cell-mediated cytotoxicity.

**Methods:**

CIK cells were generated from peripheral blood of patients affected by different B-cell malignancies using a blinatumomab-based cell culture protocol. Effector cells were combined with the anti-CD20 mAb obinutuzumab and their therapeutic activity was assessed both in vitro and in vivo.

**Results:**

CIK cells were successfully expanded in clinically relevant numbers, starting from small volumes of peripheral blood with extremely low CD3^+^ counts and high tumor burden. This relied on the addition of blinatumumab in culture, which leads to the simultaneous expansion of effector cells and the complete elimination of the neoplastic component. Moreover, CIK cells were highly cytotoxic in vitro against both B-cell tumor cell lines and autologous neoplastic targets, and had a significant therapeutic efficacy against a B-cell malignancy patient-derived xenograft on in vivo transfer.

**Conclusions:**

The combination of an easily expandable CIK cell effector population with a mAb already in clinical use establishes a tumor antigen-specific redirection strategy that can be rapidly translated into clinical practice, providing an effective therapeutic alternative for B-cell malignancies without any need for genetic modifications. Additionally, the approach can be potentially applied to an extremely vast array of different tumors by simply substituting the targeting mAb.

## Background

Aggressive B-cell malignancies are treated with a combination of chemo-immunotherapy, radiation therapy and, in the relapsed setting, with autologous stem-cell transplantation (ASCT).^1–3^ However, patients resistant to primary or salvage chemo-immunotherapy or undergoing relapse after ASCT have an extremely poor prognosis, and do not achieve long-lasting remission.^4–6^ Conversely, patients with indolent non-Hodgkin’s lymphoma or chronic lymphocytic leukemia (CLL) are usually managed with kinase inhibitors. In particular, patients failing the Bruton’s tyrosine kinase inhibitor ibrutinib and/or the B-cell lymphoma-2 (BCL2) inhibitor venetoclax are characterized by a poor prognosis and a short survival.^7–10^

In this dismal clinical context, promising therapeutic successes have been achieved in relapsed/refractory B-cell malignancies with the infusion of CD19 chimeric antigen receptor (CAR)-T cells.^11^ However, concerns have been raised on the accessibility of this therapy, as it is available only in a few centers for selected patients and at very high costs. Moreover, the infusion of CAR-T cells is often associated with severe toxicities, such as cytokine release syndrome, neurological toxicity, and persistent B cell aplasia.^12^ Besides therapy-related adverse events, relevant hurdles still dampen CAR-T cell full success and implementation, such as the technically complex manipulation process, which requires stable viral transduction, and the regulatory and financial issues.^13 14^ Nonetheless, CAR-T have critically highlighted the impact that adoptive cell immunotherapy (ACT) may have even in highly severe conditions that are not amenable to further treatments.

Many of those hindrances might be overcome whether the effector population would be easily generated in clinically relevant numbers, and could be redirected against the tumor in a target-specific manner without genetic modifications. In this regard, cytokine-induced killer (CIK) cells represent a population of effectors that can be largely expanded from peripheral blood mononuclear cells (PBMCs).^15 16^ These cells show a mixed NK/T cell phenotypical and functional profile,^17^ as they express both CD3 and CD56, and exert an MHC-unrestricted antitumor activity against a broad range of tumor histotypes, without requiring prior antigen exposure or priming.^18 19^ CIK cells have already been extensively evaluated in both preclinical and clinical studies^20–26^; these latter, in particular, have demonstrated the feasibility, the therapeutic efficacy and the very low toxicity in vivo of CIK cell infusions. Indeed, CIK cells cause very limited side effects in patients and almost completely lack graft-versus-host disease activity, even in a fully allogeneic setting, meaning that they do not induce damage in healthy tissues and hematopoietic precursors.^27^ Additionally, they can be CAR-redirected through non-viral transposon systems,^28^ and can also exert relevant antibody-dependent cell-mediated cytotoxicity (ADCC) on incubation with clinical-grade monoclonal antibodies (mAbs) due to CD16a expression.^29 30^

Here, we report that CIK cells can be successfully generated from small amounts of peripheral blood even from B-cell malignancy adult patients with extremely low CD3^+^ counts and high tumor burden, by means of a blinatumomab-based protocol that leads to both the expansion of clinically relevant numbers of effector cells and the complete elimination of the neoplastic component from cultures. Overall, we demonstrated that CIK cells exert a relevant in vitro cytotoxicity against both B-cell tumor cell lines and autologous neoplastic targets when combined with the humanized anti-CD20 mAb obinutuzumab (OBI). Besides, the combined therapy was effective in vivo against a B-cell malignancy patient-derived xenograft (PDX). Thus, the combination of an easily expandable effector population with mAbs already in clinical use could provide an effective therapeutic alternative that does not require genetic modifications. Furthermore, this approach is endowed with both antigen-specific retargeting and modularity to be applied not only in B-cell malignancies but virtually in an extremely vast array of different tumors simply based on the change of the employed mAb.

## Methods

### CIK cell generation from healthy donors

CIK cells were obtained from healthy donors as previously described.^29^ Briefly, PBMCs from buffy coats provided from the Blood Bank of Padua Hospital, Italy, were isolated by density gradient centrifugation (Lymphoprep, STEMCELL Technologies Inc, BC, Canada). Cells were resuspended in Roswell Park Memorial Institute (RPMI) 1640 (Euroclone, Milan, IT) with 10% heat-inactivated fetal bovine serum (FBS, Gibco, Thermo Fisher, Massachusetts, USA), 1% ultraglutamine, 1% HEPES buffer and 1% penicillin/streptomycin (Lonza, Switzerland) (complete RPMI) and incubated at 37°C, 5% CO_2_. At day 0, 1000 U/mL of recombinant human interferon-γ (rhIFN-γ) (R&D, MN, USA) was added to the medium, followed 24 hours later by 50 ng/mL of anti-CD3 pure human-functional grade antibody (OKT-3, Miltenyi Biotec, California, USA) and recombinant human interleukin 2 (rhIL-2) (Proleukin, Novartis, Switzerland) at 500 IU/mL. Fresh rhIL-2 was added to the medium every 3–4 days.

### CIK cell generation from patients

PBMCs were obtained from peripheral blood samples of adult patients (age, 66.6±4.4 years) and were expanded in G-Rex gas-permeable flasks (Wilson Wolf Saint Paul, Massachusetts, USA).^16^ At day 0, PBMCs were seeded in G-Rex6M at a density of 5×10^5^ cells/cm^2^ (5×10^6^ cells/well) in 40 mL of X-VIVO 10 medium (Lonza, Switzerland) supplemented with 1% Penicillin/Streptomycin (Lonza, Switzerland) and rhIFN-γ (R&D, MN, USA) at 1000 U/mL. After 24 hours, anti-CD3 pure human-functional grade antibody (OKT-3, Miltenyi Biotec, CA, USA) at 50 ng/mL and rhIL-2 (Proleukin, Novartis, Switzerland) at 500 IU/mL were added. Fresh rhIL-2 was added to the medium every 3–4 days. Where indicated, the CD3xCD19 bispecific antibody (BsAb) blinatumomab (Blina) was added to the culture at 1 ng/mL in addition to CD3 antibody (BL-CIK protocol).

### Flow cytometry

Multicolor flow cytometry was used to characterize the phenotype of all CIK cell cultures, and to evaluate CD19 and CD20 expression on both target cell lines and primary samples, using the antibodies listed in online supplemental table 1. The positivity to the markers evaluated on CIK cells was determined by gating on CD3^+^CD56^+^ cells. Naïve/memory subsets and regulatory T cells (Treg) were identified according to literature.^31–33^ Flow cytometry was performed using Celesta flow cytometer and DIVA software (BD Bioscience, California, USA), and data analyses were performed using FlowJo software (Treestar).

10.1136/jitc-2021-002475.supp1 Supplementary data

### Depletion of Natural Killer cells

Where indicated, Natural Killer (NK) cells were removed from bulk cultures by immunomagnetic depletion as already reported.^29^ CIK cells were harvested between day 14 and day 21 of culture and stained with an APC-conjugated anti-NKp46 antibody (clone 9EJ, Miltenyi Biotec, California, USA). Cells were then washed, mixed with anti-APC microbeads (Miltenyi Biotec), and separated using a LD MACS Column and MACS separator (Miltenyi Biotec) according to the manufacturer’s instructions.

### Cytotoxicity assays

Cytotoxic activity of CIK cells was assessed using a calcein-AM release assay against Raji (Burkitt lymphoma), EHEB (EBV^+^ lymphoma), Karpas-422 (Follicular lymphoma), Granta-519 (Mantle cell lymphoma), RCK-8, TMD-8, and OCI-Ly7 (diffuse large B-cell lymphoma (DLBCL), or against a single cell suspension obtained as described in online supplemental methods by digestion of PDX tissue samples. Target cells were labeled with 3.5 µM calcein-AM (Merck, Germany) in complete RPMI medium and plated at the indicated E/T ratios in presence of 1 µg/mL of the anti-CD20 antibodies rituximab (RTX), or 1 µg/mL of OBI or 1 µg/mL IgG_1_ isotype control antibody (Iso). After a 4-hour incubation at 37°C, the fluorescence released in the supernatant was measured using the VICTOR Multilabel Plate Reader (PerkinElmer, Massachusetts, USA). Each test was performed in triplicate. The results are expressed as % Specific Lysis = (experimental release – spontaneous release)/(maximal release–spontaneous release) x 100. Maximum and spontaneous releases were obtained by incubating target cells with 3% Triton X-100 (Merck, Germany) or complete RPMI medium and relative mAbs, respectively. For cytotoxicity assessment on primary samples, target cells were labeled with 5 µM of CellTrace Violet Cell Proliferation Kit (Thermo Fisher, Massachusetts, USA) for 20 min at 37°C, and incubated with CIK cells at 25:1 Effector/Target (E/T) ratio for 4 hours at 37°C. Cells were then stained with BD Horizon Fixable Viability Stain 780 (FVS780, BD Bioscience) and with Annexin V (APC, Thermo Fisher, Massachusetts, USA) according to the manufacturer instructions. The percentage of FVS780^+^ necrotic cells and AnnexinV^+^ apoptotic cells was measured in flow cytometry within the CellTrace^+^ target cells.

### In vivo studies

On day 0, 6–8 weeks old female NOD/SCID common γ chain knockout (NSG, The Jackson Laboratory, Maine, USA) mice were injected subcutaneously (s.c.) with 1×10^6^ MCL3-PDX cells (see online supplemental methods). Seven days later, when the tumors became palpable and reached a similar size in all animals, mice were randomly assigned to experimental groups. Mice were treated daily, for seven consecutive days, by intravenous coadministration of 10^7^ CIK cells resuspended with 10 mg/kg of the anti-CD20 mAb OBI (CIK +OBI group); 10^7^ CIK cells resuspended with 10 mg/kg of the isotype control mAb (CIK +Iso group); 10 mg/kg of OBI (OBI group), or left untreated. Tumor growth was monitored by caliper measurement and the volume was calculated using the formula: Tumor volume (mm^3^)=D×d^2^/2, where D and d are the longest and the shortest diameters, respectively. Mice were sacrificed when they showed signs of suffering, such as weight loss, ruffling of hair or difficulties in movements, and when subcutaneous masses exceeded a 800 mm^3^ volume or appeared ulcerated.

### Multiplex fluorescence immunohistochemistry

Multiplex fluorescence immunohistochemistry (mIHC) analysis was carried out on 4 µm thick formalin fixed and paraffin embedded (FFPE) tissue sections from MCL3-PDX samples, using the Opal 7-colors manual IHC kit (PerkinElmer). The slides were stained with the combinations of primary antibody-Opal tyramide signal amplification (TSA) reported in online supplemental table 2, and with Spectral DAPI (FB1490, Perkin Elmer, Massachusetts, USA). Stained slides were scanned at 20X using the Mantra Quantitative Pathology Workstation (PerkinElmer), and analyzed with InForm 4.9 Image Analysis Software (V.2.4.2, PerkinElmer). Results are represented as the mean of cell counts/mm^2^ of all analyzed slides for each experimental group. An average of 80 snaps was collected for each experimental group.

### Statistics

Statistical significance was analyzed by multiple t-test or two-way analysis of variance test with Bonferroni’s correction for multiple comparisons, as specified in figure legends. Mice survival was compared using log-rank survival statistics. Histograms represent mean value ±SD; box plots represent 25th and 75th percentiles and median value, and their whiskers go from minimum to maximum values. Statistical analysis was performed using GraphPad Prism V.7 software (*p<0.05, **p<0.01, ***p<0.001 and ****p<0.0001).

## Results

### The combination of CIK cells and anti-CD20 OBI has high killing activity against B-cell malignancies

**Figure 1:**
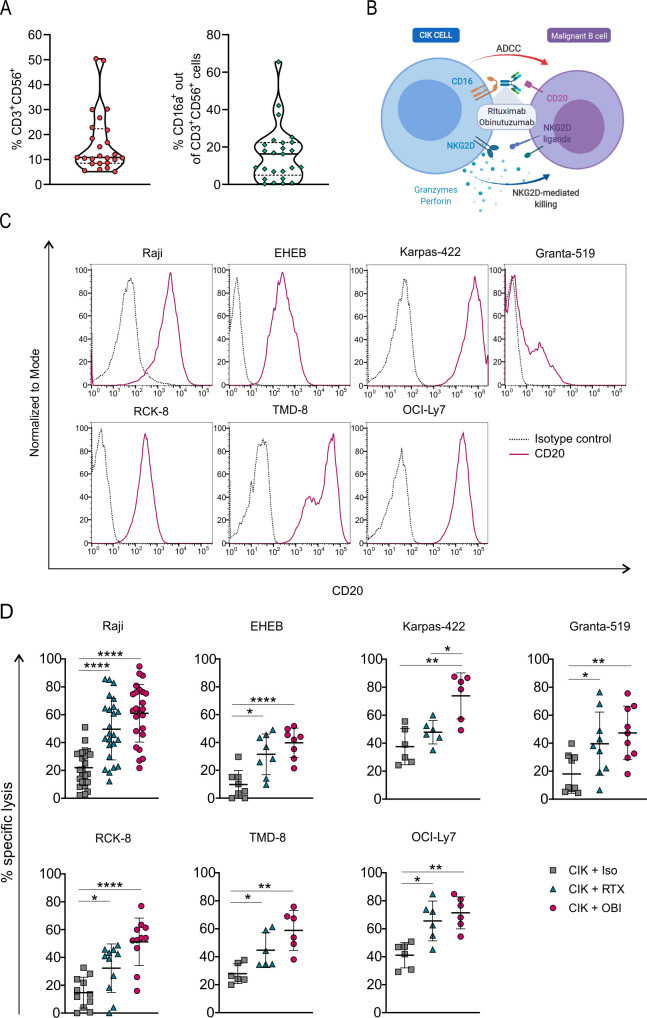
CIK cell cytotoxicity is increased by the combination with anti-CD20 mAbs. (A) Violin plots showing 25th and 75th percentiles and median value of the percentage of CD3^+^ CD56^+^ CIK cells and CD16a^+^ CIK cell subsets within day 14 and 21 of cultures generated from healthy donors (n=25). (B) Graphical representation of the CIK+ mAbs combination strategy to target and kill B-cell malignancies (created with BioRender.com). (C) CD20 expression on Raji (Burkitt lymphoma), EHEB (EBV^+^ lymphoma), Karpas-422 (follicular lymphoma), Granta-519 (Mantle cell lymphoma), RCK-8, TMD-8, and OCI-Ly7 (diffuse large B-cell lymphoma) malignant B-cell lines. (D) Cytotoxicity of CIK cells in combination with an irrelevant antibody (CIK +Iso, *squares*) or with the anti-CD20 mAbs rituximab (CIK +RTX, *triangles*) or obinutuzumab (CIK +OBI, *circles*), against the B-cell tumor lines. Lytic activity was measured by a 4-hour calcein-AM release assay performed between days 14 and 21 of CIK cell cultures. The symbols refer to the specific killing of individual CIK cell cultures from different donors at an E/T ratio of 25:1, and mean values±SD (n=6–22) are reported. Data were analyzed by multiple t-test (*p<0.05, **p<0.01, and ****p<0.0001). ADCC, antibody-dependent cell-mediated cytotoxicity; CIK, cytokine induced killer; mAbs, monoclonal antibodies; OBI, obinutuzumab; RTX, rituximab.

PBMCs from healthy donors were stimulated with IFN-γ, IL-2 and CD3 antibody to generate CIK cell cultures. The resulting populations consisted of 16.7%±12.4% CD3^+^CD56^+^ cells, and the CD3^+^CD56^+^CD16a^+^ subset was 16.2%±15.2% (figure 1A). CIK cells were combined with two anti-CD20 antibodies, namely RTX and OBI (figure 1B), and challenged in vitro against a panel of seven CD20-expressing B-cell lines (figure 1C). CIK cells exhibited a limited cytotoxicity when used in combination with an irrelevant control antibody (isotype antibody, Iso), but disclosed a strong ADCC activity when admixed with either anti-CD20 mAbs. In particular, OBI induced a significant increase of cytotoxicity against all cell lines, while RTX significantly improved lysis in six out of the seven CD20^+^ targets (figure 1D). These data are in line with the superior ADCC activity described for OBI as compared with RTX.^34 35^ RTX or OBI alone did not induce any target cell lysis, as the values of spontaneous release in the presence or absence of mAbs were comparable (data not shown).

At the end of the expansion, CIK cell bulk cultures always comprised a negligible percentage of NK cells (online supplemental figure 1A). To dissect the relative contribution of such component and of CIK cells in the ADCC phenomena, NK cells were completely removed by immunomagnetic depletion (online supplemental figure 1B, C). When challenged against target cells, the activity of the NK-depleted effectors was not impaired (online supplemental figure 1D). Additionally, the enhancement of cytotoxicity induced by RTX and OBI remained evident also after NK cell depletion, thus confirming our previous observations in the context of solid tumors^29^ that the mAb-mediated improved killing is accountable to the CD3^+^CD56^+^ cell fraction within the bulk culture.

**Figure 2:**
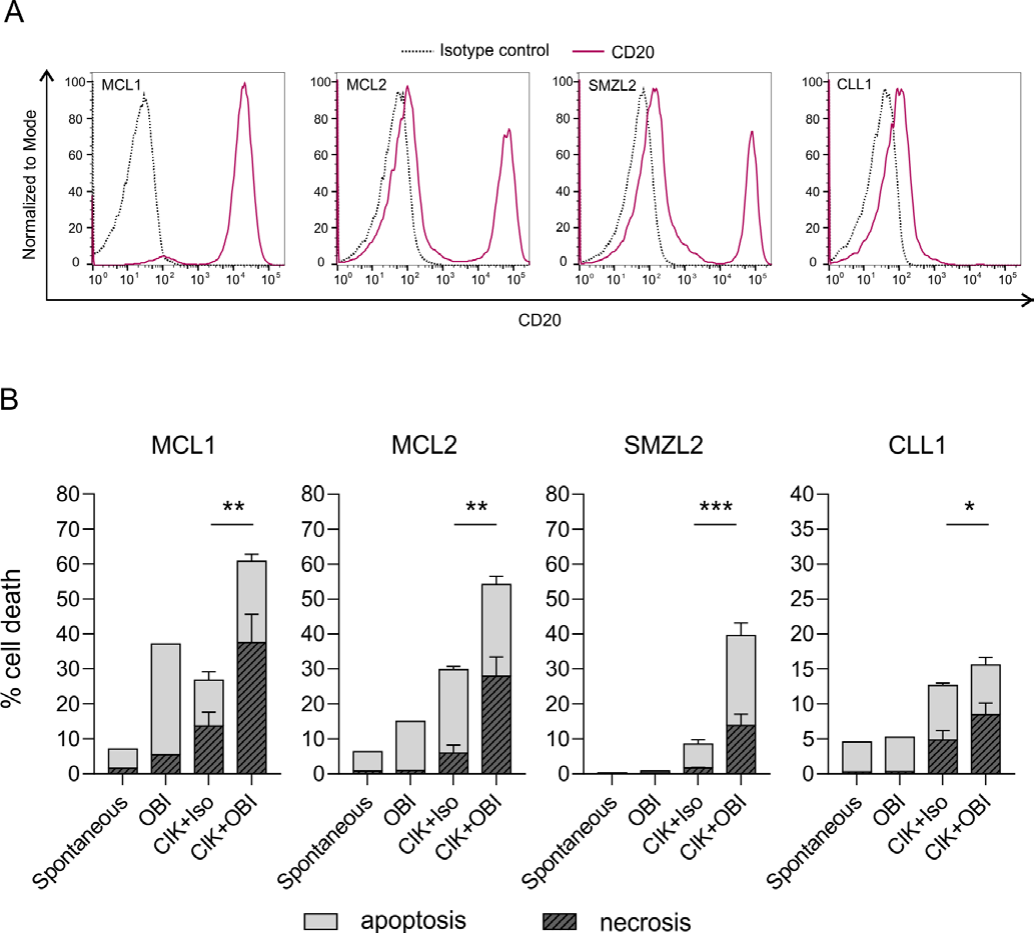
CIK cells in combination with obinutuzumab are highly effective against primary tumor samples. (A) CD20 expression on PBMCs obtained from patients affected by mantle cell lymphoma (MCL1, CD20^+^ cells=91.6%; MCL2, CD20^+^ cells=30.0%), splenic marginal zone lymphoma (SMZL2, CD20^+^ cells=37.6%) and chronic lymphocytic leukemia (CLL1, CD20^+^ cells=11.2%). (B) Percentage of cell death induced on target PBMCs that were left untreated (spontaneous), incubated with OBI only or with CIK cells from healthy donors in combination with an isotype antibody (CIK +Iso) or with obinutuzumab (CIK +OBI). The cytotoxicity assay was carried out at an E/T of 25:1 (n=3). The percentage of cell death (apoptosis/necrosis) was evaluated by flow cytometry within cell Trace-positive target cells on staining with fixable viability stain (FVS780) and annexin V (ANN V). Apoptotic cells were defined as FSV^–^/Ann V^+^, while necrotic cells were either FVS^+^/Ann V^+/−^. CIK +OBI group was compared with CIK +Iso by multiple t-test (*p<0.05, **p<0.01, ***p<0.001). CIK, cytokine-induced killer; OBI, obinutuzumab; PBMCs, peripheral blood mononuclear cells.

Based on all these results, OBI was selected as the most performant mAb for subsequent studies. In particular, we first assessed the therapeutic potential of healthy donor CIK cells+ OBI combination strategy against primary tumor samples. PBMCs were freshly isolated from the blood of patients with different levels of malignant B cells in the peripheral blood (table 1), evaluated for CD20 expression by flow cytometry (figure 2A), and used as targets for CIK cells. On incubation with OBI-added CIK cells, all primary specimens underwent an almost complete killing, considering the percentage of malignant cells present in the sample. The killing of target cells was the result of a combination of apoptosis and necrosis, which was target-specific since the addition of an irrelevant control mAb produced only marginal effects. Notably, OBI alone had a negligible impact mainly based on the induction of apoptosis-mediated cell death (figure 2B).

**Table 1:** Clinical features of B-cell lymphoma patients

Patient	Disease	State at enrollment	Tumor features	Previous treatment
**CLL1**	Chronic lymphocytic leukemia (CLL)	Relapsed	Stage IV Rai. High-risk features, including unmutated IGHV gene status, complex karyotype, TP53 disruption (both deletion and mutation).	Ibrutinib failure.
**CLL2**	CLL	Relapsed	Stage IV Rai. Unfavorable prognostic markers including complex karyotype and TP53 mutation.	Several lines of treament: fludarabine-cyclophosphamde-rituximab, twice, rituximab single agent, BR (bendamustine-rituximab), ibrutinib, venetoclax.
**CLL3**	CLL	Naïve	Stage II Rai. Favorable prognostic variable, including mutated status of IGHV gene, normal FISH and karyotype, absence of TP53 mutation.	–
**CLL4**	CLL	Naïve	Stage II Rai. Favorable markers (mutated conformation of IGHV, 13q14 deletion and absence of both TP53 deletion or mutation).	–
**MCL1**	Mantle cell lymphoma (MCL)	Naïve	Stage IV. Absence of TP53 mutation	–
**MCL2**	MCL	Relapsed	Stage IVB blastoid variant, t(11;14) +.	Three alternating cycles of R-CHOP21 with R-DHAOx (total cycles n=6) and autologous stem cell transplantation.Ibrutinib failure.
**SMZL1**	Splenic marginal zone lymphoma (SMZL)	Naïve	Stage IV	–
**SMZL2**	SMZL	Relapsed	Stage IVA, extranodal localization.	Splenectomy, BR, ibrutinib and venetoclax.
**B-ALL1**	Early B cell precursor acute lymphoid leukemia (B-ALL)	Relapsed	High-risk according to 2016 WHO classification: t(t;11) (4q21;11q23).	Prephase: cyclophosphamide and prednisone. I cycle: idarubicin, dexamethasone, vincristine e peg- asparaginase. II cycle: idarubicin, cyclophosphamide, cytarabine, 6-mercaptopurine, dexamethasone. Intrathecal therapy with methotrexate, cytarabine and dexamethasone. III cycle: methotrexate and cytarabine. At first relapse received dexamethasone for 2 days and vincristine for 1 day as debulking, followed by blinatumomab according to conventional doses and schedule. Blood sample was taken at the second relapse.

IGHV, immunoglobulin heavy chain variable.

### Fully functional CIK cells can be generated from high tumor burden patients using blinatumomab with elimination of contaminant tumor cells

**Figure 3:**
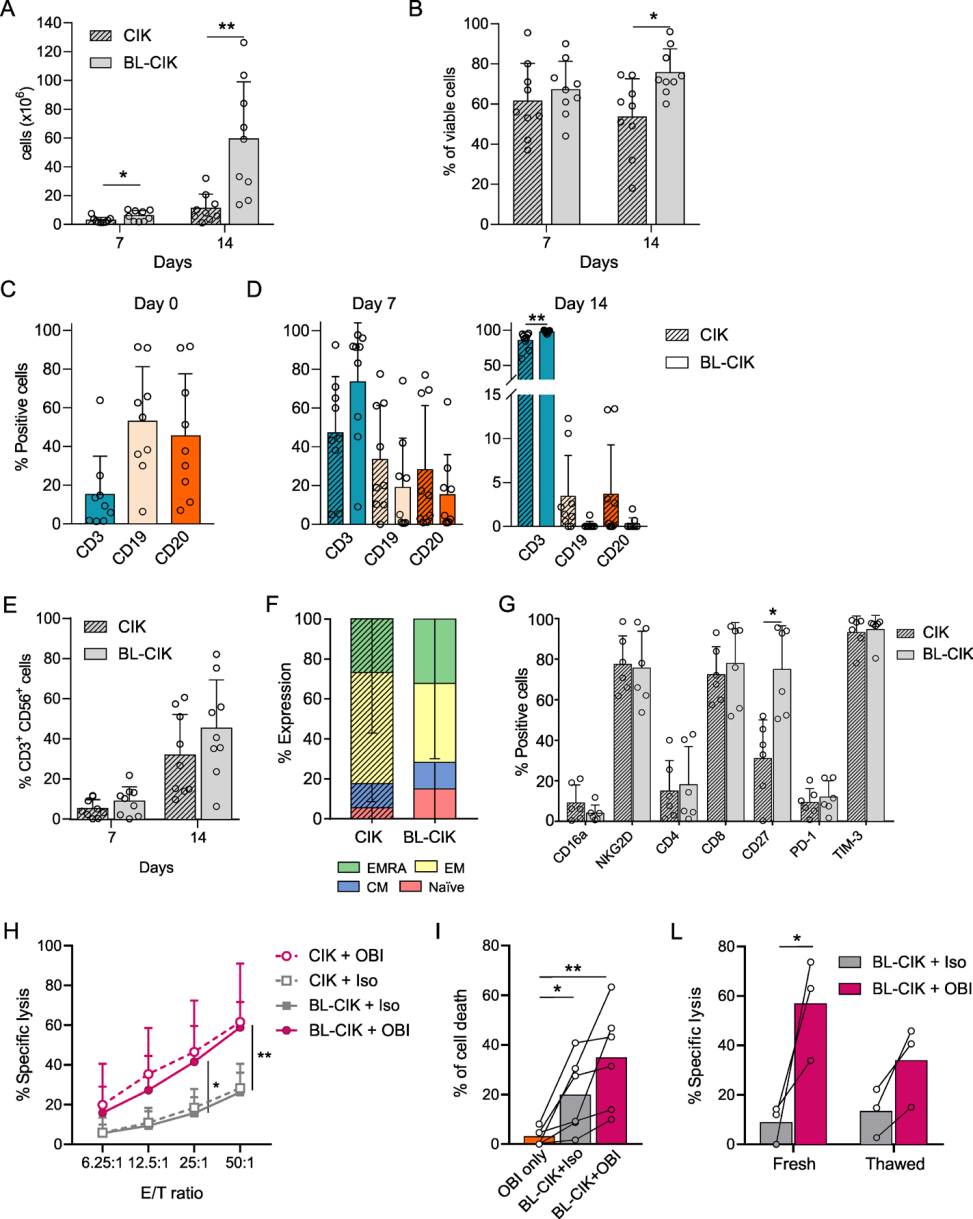
Expansion and characterization of CIK cells from PBMCs of B-cell lymphoma patients. (A) CIK cell expansion with the standard protocol (*crossed bars*, CIK) or with the addition of Blina at day 1 (*solid bars*, BL-CIK). Total cell numbers were evaluated at day 7 and 14 of culture. (B) Cell viability was calculated as the percentage of viable cells at the two different time points. (C) Percentages of CD3^+^, CD19^+^ and CD20^+^ cells were assessed at day 0, and (D) 7 and 14 days later (n=9). Data were analyzed by multiple t-test (*p<0.05, **p<0.01). (E) Percentages of CD3^+^CD56^+^ CIK cells within the bulk cultures expanded with the standard protocol (*crossed bars*, CIK) or with the addition of Blina (solid bars, BL-CIK) (n=9). (F) Comparison of the naïve/memory phenotype at day 14 of culture. Cells were stained with CD62L and CD45RA to identify the naïve (naïve, CD62L^+^CD45RA^+^), central memory (cm, CD62L^+^CD45RA^-^), effector memory (EM, CD62L^−^CD45RA^−^), and EmrA (effector memory Ra^+^, CD62L^−^CD45RA^+^) subsets within the CD3^+^CD56^+^ population (n=6). (G) Expression of different markers within the CD3^+^CD56^+^ subset at day 14 of culture (n=6). Data were analyzed by multiple t-test (*p<0.05, **p<0.01). (H) CIK and BL-CIK cells were challenged against Raji tumor cell line in combination with obinutuzumab or isotype antibody. Results show mean values±SD of specific lysis at different E/T ratios. Lytic activity was measured by calcein-AM release assay at day 14 of culture (n=6). (I) Percentage of cell death induced on patient PBMCs after 4 hour incubation with obinutuzumab only, or autologous BL-CIK cells in combination with isotype antibody (BL-CIK +Iso) or obinutuzumab (BL-CIK +OBI) at an E/T 25:1 (n=6). (L) Cytotoxicity of fresh or thawed BL-CIK cells challenged against Raji tumor cells at an E/T 25:1 (n=3). Data were analyzed by two-way ANOVA with Bonferroni correction (*p<0.05, **p<0.01). ANOVA, analysis of variance; CIK, cytokine induced killer; PBMCs, peripheral blood mononuclear cells.

To assess the therapeutic potentiality in an autologous setting where patients often present strong lymphopenia and a high tumor burden, we tested an original protocol of CIK cell expansion that employs Blina. Effector cells were expanded from PBMCs of nine patients affected by different types of B-cell malignancies, both at diagnosis and after relapse (table 1). Thawed (n=2) or freshly isolated (n=7) PBMCs (table 2) were plated in serum-free medium in G-Rex devices,^16^ and stimulated with IFN-γ, IL-2, and either CD3 mAb only (CIK, standard protocol) or the combination of CD3 mAb and Blina (BL-CIK cells, CD3+ Blina protocol). Already after 1 week, BL-CIK cells showed an enhanced expansion compared with CIK cells cultured with the standard protocol, and such difference became even more pronounced at day 14. Indeed, starting from a small number of PBMCs (5×10^6^ cells), the cell yield after 2 weeks of culture was 59.3±39.7×10^6^ and 11.2±9.8×10^6^ cells for BL-CIK and standard protocol cultures, respectively (figure 3A), with a mean fold increase of 11.9 vs only 2.5 (table 2). Interestingly, BL-CIK cultures exhibited also a significantly improved viability (figure 3B).

**Table 2:** Yields of CIK cell expansion from PBMCs obtained from B-cell lymphoma patients

		Day 0						Day 14					
		State	WCC (x10^9^/L)	Blood volume (mL)	Total no of PBMCs (x10^6^)	Total no of CD3^+^ cells (x10^6^)	Seeded cells (x10^6^)	BL-CIK			CIK		
								No of cells harvested (x10^6^)	Fold increase	Hypothetical yield of cells (x10^6^)^*^	No of cells harvested (x10^6^)	Fold increase	Hypothetical yield of cells (x10^6^)*
**Patient**													
**CLL1**	Relapsed	Fresh	49.3	18	91.0	7.2	5	67.4	13.5	1226.7	32.0	6.4	582.4
**CLL2**	Relapsed	Fresh	5.2	15	42.0	1.3	5	103.5	20.7	869.4	5.9	1.2	49.6
**CLL3**	Naïve	Fresh	50.7	15	330.0	2.9	5	84.1	8.4	2775.3	5.5	1.5	478.5
**CLL4**	Naïve	Fresh	61.2	18	420.0	0.8	5	29.6	14.8	6216.0	7.9	3.7	1541.4
**MCL1**	Naïve	Thawed	336.3	18	3200.0	5.0	5	59.1	11.8	37 824.0	1.1	0.2	704.0
**MCL2**	Relapsed	Fresh	23.6	15	322.0	2.2	5	13.7	2.7	882.3	3.5	0.7	225.4
**SMZL1**	Naïve	Thawed	27.0	18	590.0	0.5	5	126.3	25.3	14 903.4	14.2	2.8	1675.6
**SMZL2**	Relapsed	Fresh	24.1	18	300.0	3.3	5	33.2	6.6	1992.0	20.6	4.1	1236.0
**B-ALL**	Relapsed	Fresh	23.0	15	100.0	14.7	5	16.6	3.3	332.0	10.1	2.0	202.0
	Mean		66.7	16.7	599.4	4.2		59.3	11.9	7446.8	11.2	2.5	743.9
	SD		102.6	1.6	990.8	4.5		39.7	7.6	12 283.8	9.8	2.0	600.9
	Median		27.0	18.0	322.0	2.9		59.1	11.8	1992.0	7.9	2.0	582.4

*Yield calculated considering the total number of PBMCs obtained from the sample.

B-ALL, B cell precursor acute lymphoid leukemia; CIK, cytokine induced killer; CLL1, chronic lymphocytic leukemia; MCL1, Mantle cell lymphoma; PBMCs, peripheral blood mononuclear cells; SMZL2, splenic marginal zone lymphoma; WCC, white cell count.

At day 0, samples from most patients presented an extremely high percentage of CD19^+^ and CD20^+^ tumor cells (mean 52.9%±28.2% and 45.5%±32.1%, respectively; figure 3C and online supplemental table 3), which progressively decreased at day 7 in either culture conditions (figure 3D). Noteworthy, a single addition of Blina at the beginning of culture succeeded to completely eradicate CD19^+^ and CD20^+^ cells at day 14. Conversely, the cells expanded in the presence of CD3 mAb only maintained a residual percentage of tumor cells, which accounted for more than 10% of the live final population in two of the patients samples (figure 3D and online supplemental table 3). Equally important, the addition of Blina allowed a significantly increased expansion of CD3^+^ cells from 15.2±19.8% to 97.4±2.1%, whereas in the standard protocol the resulting CD3^+^ component was 85.1%±12.0% at day 14 of culture (figure 3C, D). Moreover, BL-CIK cells comprised a higher percentage of CD3^+^CD56^+^ cells (figure 3E) and a slightly increased amount of naïve cells (figure 3F), as compared with the standard culture. Notably, the percentage of Tregs in the 14-day bulk cultures was irrelevant and even lower than at day 0, indicating that this expansion protocol does not foster Treg proliferation (online supplemental figure 2). Several additional markers were evaluated on the effector populations resulting from either protocol of expansion. In particular, the expression levels of CD16a, NKG2D, CD4, CD8, PD-1 and TIM-3 turned out comparable (figure 3G), and were in line with results obtained from healthy donors (online supplemental figure 3). On the other hand, a significantly increased expression of the in vivo persistence marker CD27 was detected in BL-CIK cells as compared with standard CIK cells (74.8%±21.6% and 31%±19.9%, respectively; figure 3G).

For functional characterization, BL-CIK and standard CIK cells were challenged against the CD20^+^ Raji cell line (figure 3H). Similar to CIK cells obtained with the standard protocol, BL-CIK population not only exhibited a comparable basal cytotoxicity but was also efficiently retargeted by the combination with OBI, leading to a significant increase of lysis from 26.2±9.8% to 58.9±12.8% at an E/T ratio of 50:1. Most importantly, such killing improvement was also evident when BL-CIK cells were challenged in an autologous setting. Indeed, BL-CIK combined with OBI very efficiently lysed patient PBMCs that were not susceptible to OBI alone (figure 3I). Additionally, thawed BL-CIK cells showed a remarkable cytotoxicity, demonstrating functionality even following cryopreservation (figure 3L).

In an attempt to test the potentiality of CIK cell generation even in severely ill patients, cultures were set up from PBMCs of patients affected by DLBCL, which is characterized by the absence of malignant cells in the peripheral blood, a low white cell count count and a very poor prognosis (online supplemental tables 4 and 5). Cell yields (online supplemental figure 4A), final percentages of effectors (online supplemental figure 4B) and functionality (online supplemental figure 4C, D) of either CIK or BL-CIK cell cultures were essentially similar. In particular, both effectors disclosed a strong improvement in cytotoxicity against both Raji and the DLBCL cell line RCK-8 when combined with OBI. Taken together, all these data strongly support the concept that CIK cell effectors can be easily expanded also from severely ill patients, and are efficiently retargeted by OBI to improve their therapeutic potential against B-cell tumors.

### The combined therapy with CIK cells and OBI restrains the growth of an aggressive patient-derived lymphoma xenograft

**Figure 4:**
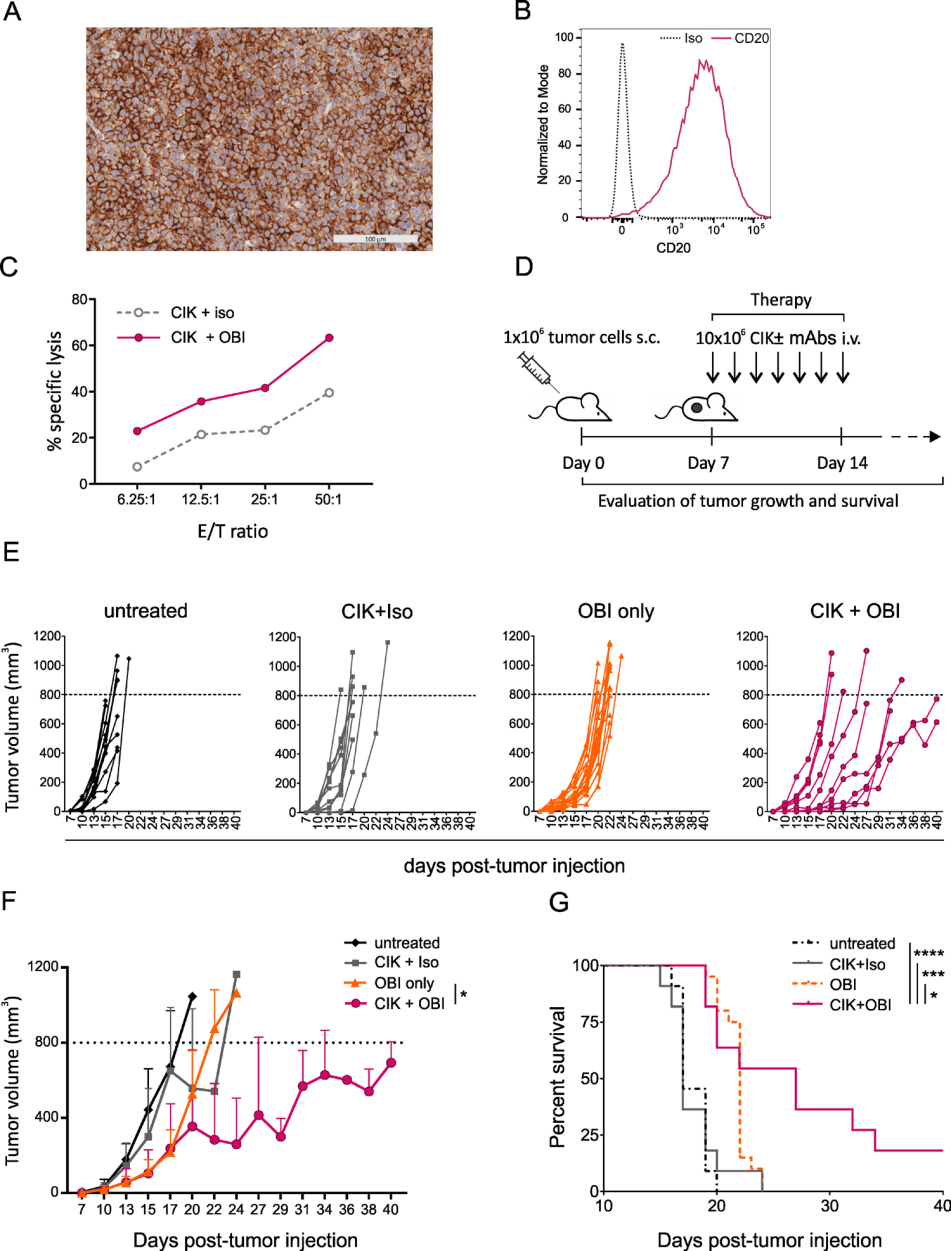
In vivo immunotherapy with CIK cells combined with OBI against an aggressive patient-derived lymphoma xenograft. (A) Representative image of anti-CD20 IHC staining (brown) of an established MCL3-PDX. (B) Flow cytometry analysis of CD20 expression on tumor cells from a digested MCL3-PDX. (C) In vitro lytic activity of CIK cells used for in vivo therapy against target cells from a digested MCL3-PDX measured by a 4-hour calcein-AM release assay. (D) Schematic representation of the in vivo therapeutic schedule. Seven days after s.c. injection of 1×10^6^ MCL3-PDX tumor cells, mice were either left untreated (n=11) or injected daily intravenous for 7 days with OBI only (n=12), or 1×10^7^ CIK cells in combination with an irrelevant antibody (CIK +Iso, n=11) or obinutuzumab (CIK +OBI, n=11). (E) Tumor growth was monitored by caliper measurement at different time points, and reported individually for each experimental group. (F) Time course of tumor growth presented as mean±SD for each experimental group. Data were analyzed by multiple t-test (*p<0.05). (G) Kaplan-Meyer survival curves of PDX-bearing mice. Statistical analysis was performed using the log-rank (Mantel-Cox) test. (*P<0.05, ***p<0.001). CIK, cytokine-induced killer; IHC, immunohistochemistry; i.v., intravenous; mAb, monoclonal antibodies; OBI, obinutuzumab; PDX, patient-derived xenograft; s.c, subcutaneously.

The therapeutic efficacy of CIK +OBI combination therapy was assessed in vivo in a mouse model of CD20^+^ PDX established from the PBMCs of an individual affected by mantle cell lymphoma in an advanced leukemic phase at diagnosis (MCL3-PDX; Online supplemental figure 5A). The analysis of CD20 expression by both IHC on FFPE tissue and flow cytometry on dissociated tumor (figure 4A, B) and the evaluation of the somatic hypermutation status of immunoglobulin heavy chain variable gene of the PDX (online supplemental figure 5B), confirmed the phenotypic and genetic similarity with the original tumor. CIK cells from a healthy donor exerted a strong in vitro cytotoxicity against PDX-derived tumor cells when combined with OBI (figure 4C), and were used in vivo to treat PDX-bearing mice. Seven days after the s.c. injection of tumor cells, mice were divided into four experimental groups (untreated, CIK +Iso, OBI only, CIK +OBI) and treated accordingly (figure 4D). Mice receiving the CIK +OBI combination therapy showed a remarkable delay in tumor growth and a significantly reduced tumor size when compared with mice treated with OBI alone (figure 4E, F). Conversely, the CIK +Iso treatment produced only marginal effects, thus indicating that the synergy between the CD20-specific antibody and CIK cells is required to enhance the antitumor effect (figure 4E, F). Indeed, only the CIK +OBI combined therapy significantly prolonged the survival of treated mice, while all other treatments were inefficient (figure 4G).

**Figure 5:**
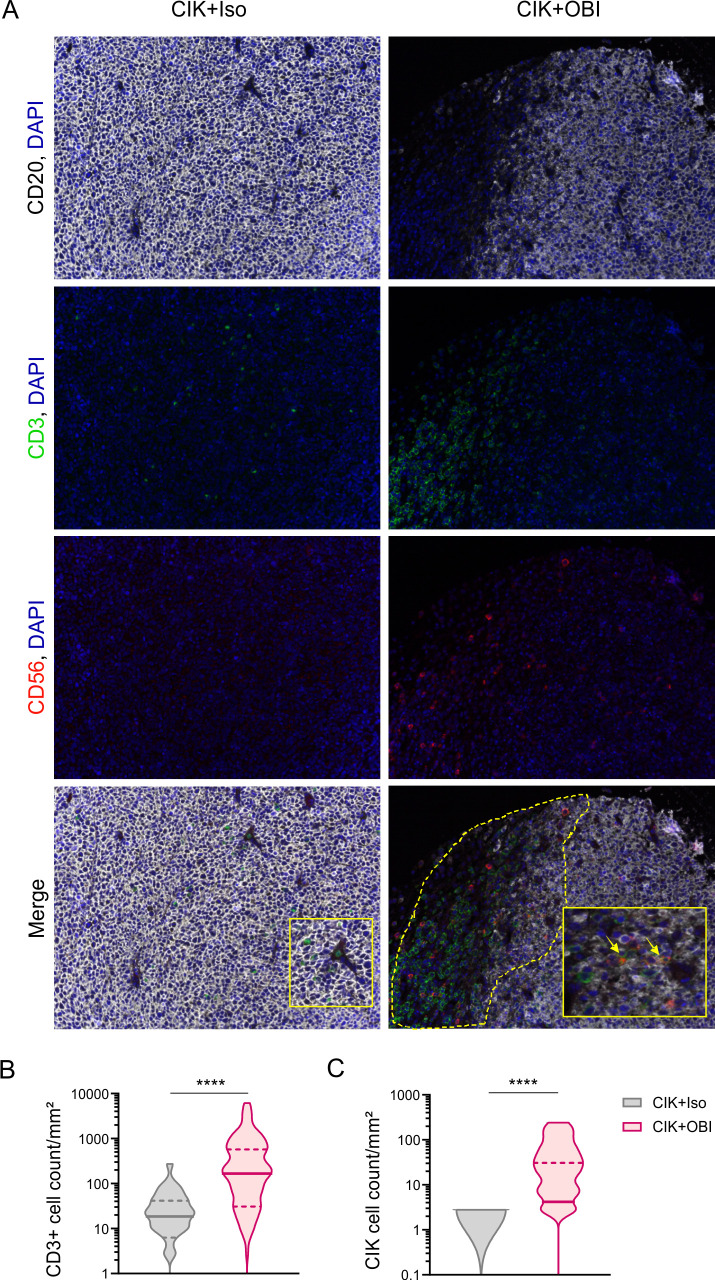
Evaluation of tumor infiltrating CIK cells by mIHC. (A) Representative figure (x20) of FFPE tumor samples collected at the sacrifice of mice, stained with CD3 (*green*), CD56 (*red*), CD20 (*white*) and nuclei (*blue*), and analyzed by mIHC. The yellow arrows indicate CD3^+^CD56^+^ cells. (B) Quantification (cell count/mm^2^) of tumor infiltrating immune cells is presented as median and quartiles of CD3^+^ and (C) CD3^+^CD56^+^ subsets detected in an average of 80 different snaps collected from each experimental group (****p<0.0001). CIK, cytokine induced killer; FFPE, formalin fixed and paraffin embedded; mIHC, multiplex fluorescence immunohistochemistry; OBI, obinutuzumab.

Tissue sections from treated mice were stained with CD3/CD56/CD20/DAPI and analyzed by mIHC (figure 5A). Samples showed an enhanced immune infiltration when CIK cells were administered in combination with OBI, as compared with mice treated with CIK +Iso. In particular, mice receiving CIK +OBI showed a significantly higher density of both CD3^+^ cells (31.91±48.87 cell counts/mm^2^ in CIK +Iso vs 503±943.7 cell counts/mm^2^ in CIK +OBI; figure 5B) and CD3^+^CD56^+^ CIK cells (0.13±0.6 cell counts/mm^2^ in CIK +Iso vs 28.51±52.85 cell counts/mm^2^ in CIK +OBI; figure 5C) within the tumor mass.

## Discussion

CD19 CAR-T are currently the most advanced T cell therapy tested in clinical trials, and have demonstrated unprecedented efficacy. Indeed, available data show high rates of remission in patients affected by certain relapsed/refractory B-cell malignancies.^13 36 37^ Eastern Cooperative Oncology Group performance status is often used to determine patient eligibility for CAR-T cell therapy. Patient selection is also based on previous treatments, comorbidities, conserved renal and hepatic function, absence of central nervous system localization, as well as adequate absolute lymphocyte count.^38^ Moreover, successful CAR-T cell manufacturing requires an adequate supply of functional T cells since prior treatments with lymphotoxic agents may have an impact on T cell function. Given the numerous exclusion criteria, a remarkable percentage of patients is not eligible for CAR-T therapy and does not have any further treatment perspective.^39^

Here, we provide evidence that tumor-specific mAb-retargeted CIK cells may represent not only a valuable option for such patients, but could also constitute a reliable alternative to CAR-T. Indeed, besides being recognized as a very promising population of effector cells for adoptive immunotherapy of cancer,^20^ their activity can be further fostered by the engagement of the CD16a receptor by tumor antigen-specific mAbs, leading to an enhanced cytotoxicity by ADCC.^29 30^

In this study, we exploited this concept in the context of B-cell hematological malignancies by combining CIK cells with two clinically relevant anti-CD20 antibodies, namely RTX and OBI. Overall, mAb-redirected CIK cells showed a strong improvement in lytic activity that was always more pronounced with OBI, consistently with the higher affinity for CD16a of its glycoengineered Fc domain.^34^ These results found a strong confirmation in vivo in an aggressive PDX model derived from a refractory/relapsed patient, where the CIK +OBI combination restrained tumor growth favoring the immune infiltrate, and prolonged survival. Although we cannot completely rule out that in the clinical setting the final outcome could depend also on additive effects between CIK-mediated ADCC and OBI alone, nonetheless such activities act both on the same therapeutic direction. Thus, our data strongly support the application of CIK cells and OBI combination for the treatment of B-cell malignancies. This consideration also relies on recent data collectively remarking the safety profile of CIK cells even after CAR retargeting^28 40^ together with the high tolerability of OBI as a monotherapy.^41 42^

In Europe, CIK as well as CAR-T cells are defined Advanced Therapy Medicinal Products (ATMP).^43^ In particular, CIK cells fall under the definition of ‘somatic-cell therapy medicinal products’ (Commission Directive 2009/120/EC), and must be produced following Good Manufacturing Practices (GMP).^44^ In this regard, the expansion of clinically relevant numbers of effector cells is one of the main hurdles of every type of ACT. In particular, in the autologous setting, the challenge is to generate sufficient effectors starting from blood samples containing mostly tumor cells and very low CD3^+^ counts. To this aim, we have upgraded a culture protocol^16^ that was developed for an immediate translation into GMP as it employs pharmaceutical grade reagents, the elimination of animal derivatives (such as FBS) and serum-free media, according to the guidelines for ATMP production,^45^ and already allows the generation of clinically-relevant numbers of CIK cells. In the BL-CIK protocol, the early addition of Blina in the culture that simultaneously targets CD3 on effectors and CD19 on target cells, led to the concomitant expansion of CIK cells and the elimination of the malignant B cell fraction, without any magnetic selection or cell sorting at the beginning of the expansion. Conversely, cultures expanded without the BsAb had consistent residual percentages of CD19^+^ and CD20^+^ cells. Indeed, it is remarkable that a single addition of a low Blina dose at the beginning of the expansion led to the successful removal of tumor cells in just 2 weeks, whereas others reported that the BsAb had to be added every 2–4 days for about 3 weeks, to achieve the elimination of CD19^+^ CLL cells in T cell expansion culture.^46^ The complete elimination of the neoplastic component provides a double advantage. First, it avoids the risk of reinjecting tumor-contaminated effectors and increases treatment safety; second, by removing malignant cells potentially endowed with inhibitory and suppressive effects,^47–49^ CIK cell expansion and fitness are improved. Indeed, the Blina-based culture protocol allowed the expansion of functional CIK cells from all the patients, irrespective of the type of the underlying disease, and to generate clinically relevant numbers of effector cells even starting from small volumes of peripheral blood, with extremely poor CD3^+^ counts.

On the other hand, the overall impact of Blina addition to patient cultures was not limited to quantitative aspects of the final cell product, but impinged also on qualitative features. In particular, BL-CIK cells showed an increased percentage of both CD3^+^ and CD3^+^CD56^+^ subsets, and a higher proportion of naïve cells that might impact positively on the efficacy of the therapy. Notably, such cells exhibited also an increased expression of CD27, which is involved in the in vivo survival after adoptive transfer, and the generation and long-term maintenance of T cell immune responses.^31^ Moreover, in view of a GMP translation, this protocol significantly reduces the number of culture manipulations and the time required to obtain a cell product free from residual tumor cells and ready to be administered, without the need of any immunomagnetic selection or depletion procedure.

In conclusion, a combined approach with CIK cells, which can be easily generated and expanded even from patients not eligible for CAR-T therapy, and a mAb already in clinical use (OBI) can be advanced as a valuable therapeutic option for refractory/relapsed B-cell malignancies. Moreover, data support the potentiality of a ‘universal’ platform for ACT, where the cytotoxicity of CIK cells can be further improved by different antigen-specific retargeting moieties (clinical-grade mAbs, Fc-engineered mAbs, BsAb and immunoligands), depending on the tumor histotype and antigen expression, without any need to genetically modify the effector population.

## Data Availability

All data relevant to the study are included in the article or uploaded as online supplemental information.
