# Supplementary material for: Innovative therapeutic strategy for B-cell malignancies that combines obinutuzumab and cytokine-induced killer cells
Source: J Immunother Cancer. 2021 Jul 16;9(7):e002475. doi: 10.1136/jitc-2021-002475 (PMC8287629; doi:10.1136/jitc-2021-002475)
Supplement: Supplementary data [file jitc-2021-002475supp001.pdf]

SUPPLEMENTAL MATERIAL

METHODS

Cell lines and cultures

Raji (Burkitt lymphoma), EHEB (EBV$^+$ lymphoma), RCK-8, TMD-8 (Diffuse Large B-cell lymphoma) and Karpas-422 (Follicular lymphoma) cell lines were cultured in RPMI 1640 (Euroclone, Milan, IT), while Granta-519 (Mantle cell lymphoma) were kept in DMEM (Euroclone, Milan, IT); both media were supplemented with 10% heat-inactivated FBS (Gibco, Thermo Fisher, MA, USA), 1% Ultraglutamine, 1% N-2-hydroxyethylpiperazine-N'-2-ethanesulfonic acid (HEPES) buffer, 1% penicillin/streptomycin (all from Lonza, Switzerland). OCI-Ly7 (Diffuse Large B-cell lymphoma) were cultured in IMDM supplemented with 20% heat-inactivated FBS (Gibco, Thermo Fisher, MA, USA), 1% Ultraglutamine, 1% HEPES buffer, 1% penicillin/streptomycin (all from Lonza, Switzerland) and 50µM 2-mercaptoethanol (Merk, Germany). All cell lines were authenticated by STR sequences analysis.

Patient-derived tumor xenograft (PDX)

The CD20-positive PDX was established by injection of PBMCs from a patient affected by disseminated mantle cell lymphoma (MCL3-PDX). Briefly, $1x10^6$ tumor cells were injected s.c. in the flank of 6-8 week-old female NOD/SCID common γ chain knockout (NSG, The Jackson Laboratory, ME, USA) mice. When tumors reached a volume of 500 mm^3, mice were sacrificed and tumors were digested using the Tumor Dissociation Kit and the gentleMACS Octo Dissociator (MACS, Miltenyi Biotec, CA, USA) following manufacturer's instructions. Cell suspensions were freshly re-injected ($1x10^6$ tumor cells, s.c.) and expanded from mouse to mouse. Xenografts were considered established after three passages. At each passage, the PDX tumors were collected and evaluated to confirm the CD20 expression by both immunohistochemistry (IHC) on FFPE tissue using anti-human CD20 mAb (clone L26, Leica Biosystems, Germany) and flow cytometry on dissociated tumor. PDX single cell suspensions were used also as target cells in cytotoxicity assays. Moreover, DNA and RNA were extracted to analyze the mutational state of the variable heavy chains of immunoglobulins (IgVh) and to compare it to the original profile of the patient sample.

SUPPLEMENTAL TABLES

Supplemental Table 1. Antibodies used for flow cytometry

Antigen	Clone	Fluorochrome	Company
CD3	UCHT1	BV510	BD Bioscience
CD8	RPA-T8	BV421	BD Bioscience
CD4	RPA-T4	APC-H7	BD Bioscience
CD62L	DREG-56	FITC	BD Bioscience
CD45RA	HI100	PerCP	BD Bioscience
CD20	2H7	PE-CF594	BD Bioscience
CD19	HIB19	FITC	BD Bioscience
CD56	HCD56	PE	BioLegend
NKG2D	1D11	APC	BioLegend
CD27	O323	FITC	BioLegend
CD16a	3G8	FITC	BioLegend
TIM-3	F38-2E2	BV421	BioLegend
PD-1	EH12.2H7	FITC	BioLegend
CD25	2A3	PECy7	BD Bioscience
CD127	HIL-7R-M21	APC-R700	BD Bioscience
CD8	SK1	BV605	BD Bioscience
CD4	SK3	PerCP-Cy 5.5	BD Bioscience

Supplemental Table 2. Combinations of antibodies and Opal used for mIHC.

Antigen	Clone	Concentration	Company	Opal
CD56	123C3	1:30	DAKO/Agilent	TSA Cy3 (Opal 570, 1:100)
CD20	L26	1:400	DAKO/Agilent	TSA Cy 5.5 (Opal 690, 1:100)
CD3	F.7.2.38	1:400	DAKO/Agilent	TSA FITC (Opal 520, 1:100)

Pietà AD, *et al. J Immunother Cancer* 2021; 9:e002475. doi: 10.1136/jitc-2021-002475

Supplemental Table 3. Phenotype of CIK cell culture:

Patient	% CD3⁺ Day 0	% CD3⁺ Day 14 BL-CIK	% CD3⁺ Day 14 CIK	% CD19⁺ Day 0	% CD19⁺ Day 14 BL-CIK	% CD19⁺ Day 14 CIK	% CD20⁺ Day 0	% CD20⁺ Day 14 BL-CIK	% CD20⁺ Day 14 CIK	% CD3⁺CD56⁺ Day 0	% CD3⁺CD56⁺ Day 14 BL-CIK	% CD3⁺CD56⁺ Day 14 CIK
**CLL1**	14.7	96.2	93.5	65.8	0.0	1.2	11.2	0.0	0.0	nd	55.9	60.6
**CLL2**	25.0	99.0	87.9	55.0	0.0	0.0	51.3	0.0	0.0	8.0	82.1	14.9
**CLL3**	5.8	99.2	88.1	62.6	0.0	10.6	67.7	0.0	13.4	0.4	40.6	15.2
**CLL4**	1.4	94.4	91.3	36.0	1.3	2.2	21.8	0.7	2.7	nd	23.6	26.7
**MCL1**	1.5	100	59.7	91.3	0.0	12.3	91.6	0.0	3.1	0	35.1	9.7
**MCL2**	9.3	94.2	71.3	30.0	0.0	1.4	30.0	0.0	0.1	0.4	75.3	51.0
**SMZL1**	1.8	97.6	84.1	90.9	0.0	2.6	90.9	2.0	13.3	0	6.3	13.9
**SMZL2**	13.5	98.3	95.6	38.3	0.0	0.6	37.6	0.0	0.4	nd	53.1	57.4
**B-ALL**	63.8	97.3	94.4	6.4	0.0	0.0	7.0	0.0	0.0	5.7	35.6	37.4
mean	15.2	97.4	85.1	52.9	0.1	3.4	45.5	0.3	3.7	2.4	45.3	31.9
SD	19.8	2.1	12.0	28.2	0.4	4.7	32.1	0.7	5.6	3.5	24.1	20.3
median	9.3	97.6	88.1	55.0	0.0	1.4	37.6	0.0	0.4	1.4	40.6	26.7

Supplemental Table 4. Clinical features of Diffuse Large B-cell Lymphoma patients.

Patient	Disease	State at enrollment	Tumor features	Previous treatment
**DLBCL1**	Diffuse Large B-cell Lymphoma (DLBCL)	relapsed	Stage IVB, DLBCL lymphoma non-GCB	Three lines of treatment: R-CHOP, R-GDP, R-IVAC
**DLBCL2**	Diffuse Large B-cell Lymphoma (DLBCL)	relapsed	Stage IVA, DLBCL	Relapsed after R-CHOP21 and stable disease with R-DHAOx
**DLBCL3**	Diffuse Large B-cell Lymphoma (DLBCL)	relapsed	Stage IVA, double-hit DLBCL lymphoma	R-CODOX-M-IVAC

Supplemental Table 5. Yields of CIK cell expansion from PBMCs obtained from DLBCL patients.

			Day 0					Day 14						
								BL-CIK			CIK			
**Patient**		*State*	*WCC ($x10^9/L$)*	*Blood volume (mL)*	*Total number of PBMCs ($x10^6$)*	*Total number of $CD3^+$ cells ($x10^6$)*	*Seeded cells ($x10^6$)*	*Number of cells harvested ($x10^6$)*	*fold increase*	*Hypothetical yield of cells ($x10^6$)[a]*	*Number of cells harvested ($x10^6$)*	*fold increase*	*Hypothetical yield of cells ($x10^6$)[a]*
**DLBCL1**	*relapsed*	fresh	5.6	23.0	37.0	19.5	5	89.1	17.8	659.1	108.6	21.7	803.4
**DLBCL2**	*relapsed*	fresh	2.0	16.5	16.4	9.7	5	262.2	52.4	860.0	143.1	28.6	469.2
**DLBCL3**	*relapsed*	fresh	3.1	16.5	11.5	3.9	5	32.5	6.5	74.7	26.5	5.3	61.0
	mean			18.7	21.6	11.0	5	127.9	25.6	531.3	92.7	18.5	444.5
	SD			3.8	13.5	7.9	0	119.7	23.9	408.0	59.9	12.0	371.8
	median			16.5	16.4	9.7	5	89.1	17.8	659.1	108.6	21.7	469.2

[a] Yield calculated considering the total number of PBMCs obtained from the sample.

SUPPLEMENTAL FIGURES

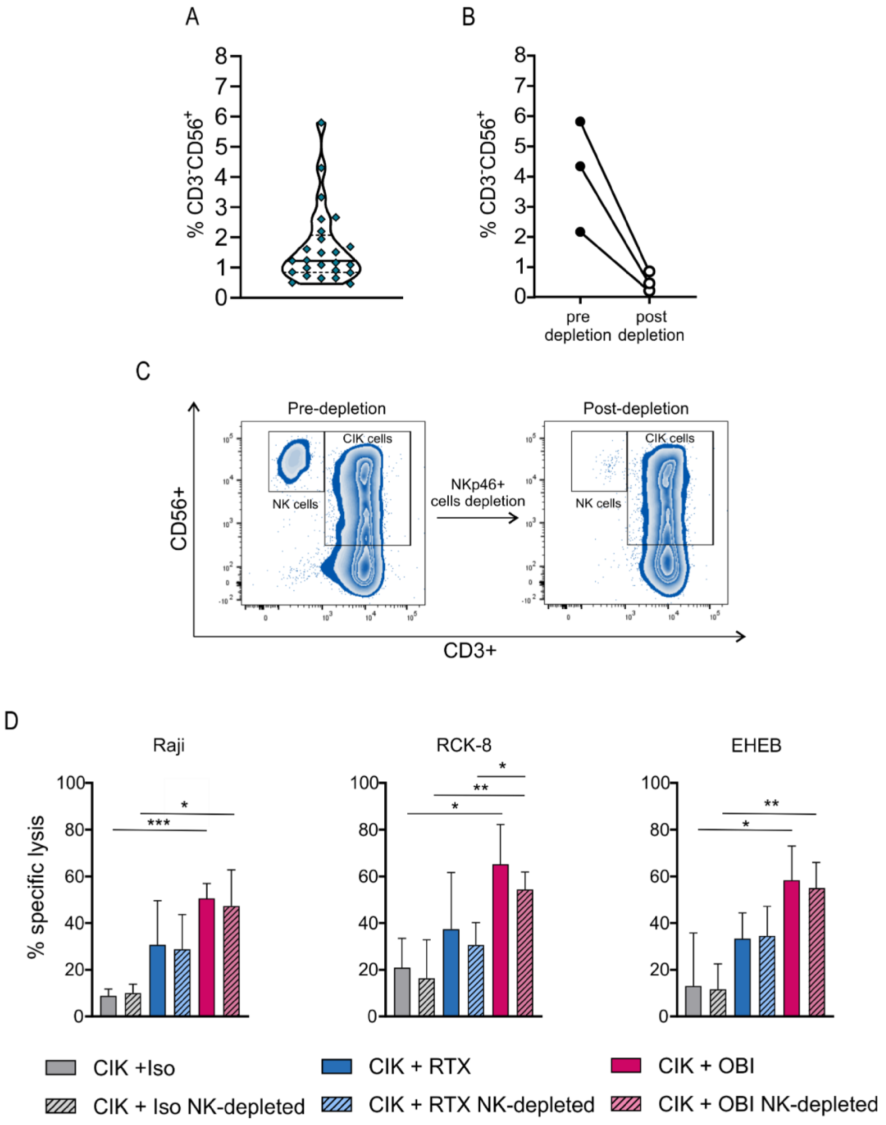

Supplemental Figure 1. Characterization of NK-depleted CIK cell cultures. (A) Violin plot showing the percentage of $CD3^{-}CD56^{+}$ NK cells within 14/21-day CIK cell bulk cultures from healthy donors (n=25). **(B)** Percentage of $CD3^{-}CD56^{+}$ NK cells before and after $NKp46^{+}$ cell depletion (n=3), as assessed by flow cytometry. **(C)** Dot plot of one representative experiment where cytometry analysis was carried out before (*left*) and after (*right*) NK depletion. **(D)** Assessment of RTX- and OBI-mediated ADCC by CIK cells before and after NK depletion; Raji, RCK-8 and EHEB tumor cell lines were used as targets. Lytic activity was measured by a 4-h calcein-AM release assay (E/T 50:1; n= 3), and bars indicate mean values ± SD. Data were analyzed by Multiple t-test (P<0.05 =*, P<0.01 =**, P<0.001 =***).

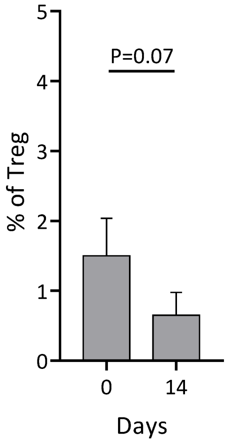

Supplemental Figure 2. Tregs in bulk cultures of B-cell lymphoma patients at days 0 and 14 of expansion. Tregs were identified as CD3$^+$/CD4$^+$/CD25$^+$/CD127$^-$ cells by multiparametric flow cytometry. Bars indicate the mean values±SD (n=5). Data were analyzed by Mann-Whitney t-test.

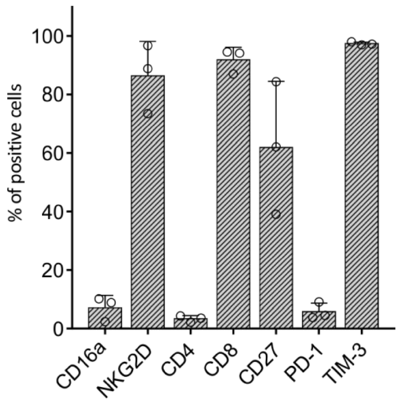

Supplemental Figure 3. Expression of CD16a, NKG2D, CD4, CD8, CD27, PD-1 and TIM-3 markers within the CD3$^+$CD56$^+$ subset of CIK cell cultures expanded from healthy donors in GMP-grade condition using the standard protocol (n=3).

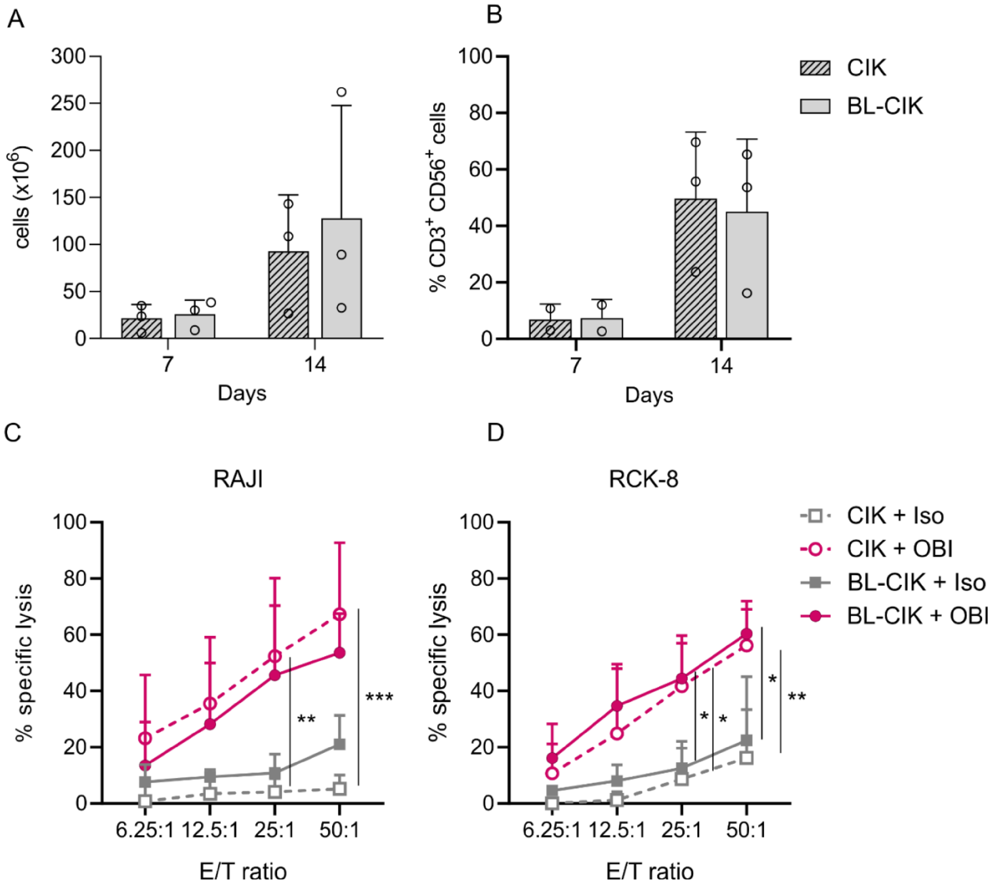

Supplemental Figure 4. Expansion of CIK cell cultures from PBMCs of DLBCL patients. **(A)** Effector cell expansion at days 7 and 14 of culture obtained with the standard protocol (*crossed bars*, CIK) or following the addition of Blinatumomab at day 1 (*solid bars*, BL-CIK). **(B)** Percentage of $CD3^{+}CD56^{+}$ cells detected in cell cultures at days 7 and 14 (n=3). **(C)** Lytic activity of CIK and BL-CIK cells against Raji and **(D)** RCK-8 tumor cell lines in the presence of obinutuzumab or an isotype antibody. Results show mean values ± SD of specific lysis at different E/T ratios. Lytic activity was measured by calcein-AM release assay at day 14 of culture (n=3). Data were analyzed by two-way ANOVA with Bonferroni correction ($P<0.05$ =*, $P<0.01$ =**).

A

Patient	Disease	State at enrollment	Tumor features	Previous treatment	% CD20$^+$
**MCL1**	Mantle cell lymphoma (MCL)	naïve	Unmutated IGHV gene status	-	97.4

B

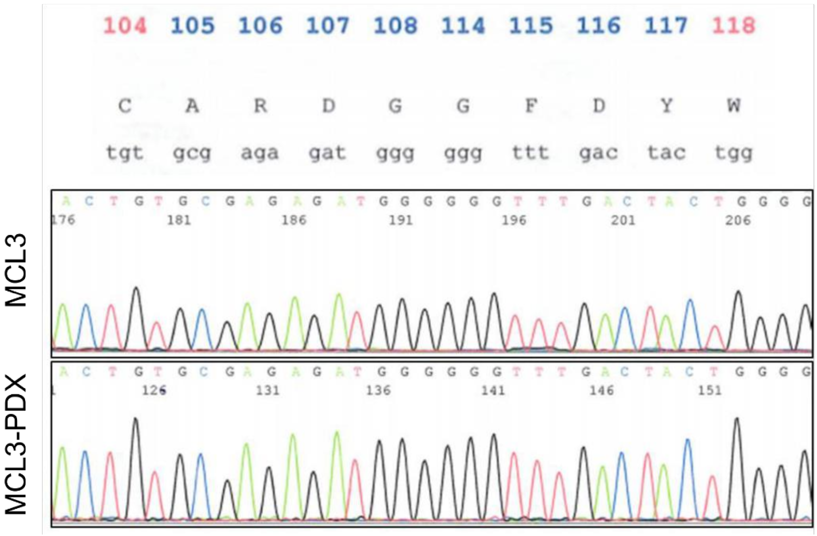

Supplemental Figure 5. Characterization of MCL3-PDX. **(A)** Clinical features of the patient from whom the MCL3-PDX was derived. **(B)** Analysis of the IgV$_H$ mutation status. Comparison between the VDJ nucleotide and the amino acid sequences of the MCL3 patient and the established MCL3-PDX.
